# Docetaxel-Loaded Disulfide Cross-Linked Nanoparticles Derived from Thiolated Sodium Alginate for Colon Cancer Drug Delivery

**DOI:** 10.3390/pharmaceutics12010038

**Published:** 2020-01-02

**Authors:** Hock Ing Chiu, Asila Dinie Ayub, Siti Nur Aishah Mat Yusuf, Noorfatimah Yahaya, Erazuliana Abd Kadir, Vuanghao Lim

**Affiliations:** 1 Integrative Medicine Cluster, Advanced Medical and Dental Institute, Universiti Sains Malaysia, Bertam, 13200 Kepala Batas, Penang, Malaysia; hockingchiu@gmail.com (H.I.C.); asiladinie@gmail.com (A.D.A.); nuraishahyusuf@unimap.edu.my (S.N.A.M.Y.); noorfatimah@usm.my (N.Y.); erazuliana@usm.my (E.A.K.); 2 Department of Chemical Engineering Technology, Faculty of Engineering Technology, Universiti Malaysia Perlis, UniCITI Alam Campus, 02100 Padang Besar, Perlis 02600, Malaysia; 3 Department of Chemistry, University of British Columbia, Vancouver, BC V6T 1Z1, Canada; 4 School of Agriculture and Food Sciences, The University of Queensland, Brisbane, St Lucia 4072, Australia

**Keywords:** disulfide cross-linked nanoparticles, thiolated sodium alginate, wheat germ agglutinin conjugation, HT-29, docetaxel

## Abstract

In this study, fluorescein-labelled wheat germ agglutinin (fWGA)-conjugated disulfide cross-linked sodium alginate nanoparticles were developed to specifically target docetaxel (DTX) to colon cancer cells. Different amounts of 3-mercaptopropionic acid (MPA) were covalently attached to sodium alginate to form thiolated sodium alginate (MPA1–5). These polymers were then self-assembled and air-oxidised to form disulfide cross-linked nanoparticles (MP1–5) under sonication. DTX was successfully loaded into the resulting MP1–5 to form DTX-loaded nanoparticles (DMP1–5). DMP2 had the highest loading efficiency (17.8%), thus was chosen for fWGA surface conjugation to form fWGA-conjugated nanoparticles (fDMP2) with a conjugation efficiency of 14.1%. Transmission electron microscopy (TEM) and scanning electron microscopy (SEM) analyses showed spherical nanoparticles, and an in vitro drug release study recorded a cumulative drug release of 48.6%. Dynamic light scattering (DLS) analysis revealed a mean diameter (MD) of 289 nm with a polydispersity index (PDI) of 0.3 and a zeta potential of −2.2 mV for fDMP2. HT-29 human colon cancer cells treated with fDMP2 showed lower viability than that of L929 mouse fibroblast cells. These results indicate that fDMP2 was efficiently taken up by HT-29 cells (29.9%). Fluorescence and confocal imaging analyses also showed possible internalisation of nanoparticles by HT-29 cells. In conclusion, fDMP2 shows promise as a DTX carrier for colon cancer drug delivery.

## 1. Introduction

Colon cancer is one of the main causes of death in Malaysia and in most countries around the world [1]. After surgical removal of a tumour, subsequent chemotherapy is often required to avoid relapse [2]. These therapies require that anticancer agents reach localised cancerous tumours in sufficient quantities to achieve effective treatment. However, the potency of these agents is limited due to a lack of effective administration routes. Most orally administered drugs are degraded or absorbed in the upper gastrointestinal tract (GIT) before reaching the targeted site [3]. Moreover, administration is non-discriminative, and both healthy and cancerous tissues are exposed to drugs, resulting in high morbidity and mortality [4]. Poor bioavailability and solubility of most anticancer drugs also limit the effectiveness of oral routes of administration [3].

Although most anticancer drugs can be delivered rectally or by intravenous injection, poor patient compliance and drug performance have been observed [5]. For instance, involvement of the non-ionic surfactant Tween 80 and ethanol [6] in the currently used intravenous administration of a DTX formulation (Taxotere^®^) lead to undesired drug distribution and pharmacokinetic profiles as well as side effects such as hypotension, neurotoxicity, myelosuppression, and epithelial necrosis [7,8,9,10]. Nevertheless, the adverse effects, which includes toxicity to normal cells can be avoided by site-specific release of drug to the colon cancer cells. pH and redox responsive with active targeting (ligands) nanocarriers can address the limitations. This would be highly beneficial in enhancing the efficacy at the site of action with less adverse effects.

Colonic drug delivery offers enhanced efficacy of anticancer drugs. It can be used to deliver drugs to treat colon diseases as well as to systemically deliver therapeutic proteins and peptides and anti-asthmatic, antihypertensive, and antidiabetic drugs [11,12]. This delivery method protects the drugs en route to the colon. Drugs are prevented from being released or degraded in the upper GIT, and they are only released and absorbed in the colon to provide a systemic drug level in the blood [13,14]. Side effects are minimised, and the high potency of the drug reduces the viability of cancerous cells [15]. Colonic drug delivery is usually performed via the oral route, as it is economical and convenient [5]. It also improves patient compliance and provides a therapy with lower dosing frequency and less expense.

Recently, reduction-responsive carriers based on disulfide bond-containing polymers have received much attention because they provide specific release of drug at the target site and respond to the physiological characteristics of the GIT [16]. Disulfide bond-containing polymers promptly degrade in cancer cells and release drug at intended site due to the reducing environment of cancer cells. The concentration of glutathione (GSH) was higher in cancerous cells than that in normal cells, providing an intracellular reductive environment for the polymer [17,18,19]. Therefore, nanocarriers derived from disulfide bond-containing polymers are stable in normal cells, in which the drug will not leak from the nanocarriers along the route to the targeted site, thus reducing the toxicity and side effects of drug to the normal cells. The increase in the bacterial population along the GIT causes a decrease in redox potential due to increasing metabolic and enzyme activities [20]. Disulfide bonds are readily reduced or cleaved in a reductive environment (low redox potential), thereby allowing the drug to be released in the colon [21,22]. This promotes the systemic delivery of drugs to the targeted part of the human body.

Polymeric nanoparticles prepared from sodium alginate may prove to be suitable carriers for delivering orally administered drugs to the colon due to their promising properties (biocompatible, bio-adhesive, biodegradable, and non-toxic) [23,24]. Nanoparticles may reduce the risk of adverse effects and long-term accumulation of the polymer in the body. The mucins contain positively charged regions (Ca^2+^), and these charges might cause alginate to bind to mucin through electrostatic adhesion and hydrogen bonding (carboxyl-hydroxyl interactions) [25]. Moreover, the binding of muco-adhesive sodium alginate with the mucins secreted by intestinal cells promotes contact between the drug and cells to prolong the length of time the drug stays at the absorption site [26]. This could increase the bioavailability and solubility of drugs and facilitate the uptake of nanoparticles.

Environmental and physiological factors in the upper GIT, such as extremely gastric pH and high intestinal enzyme activity in the stomach and small intestine, can degrade drugs [27,28]. Disulfide polymers, which survive the hostile environment of the upper GIT and are only degraded in the reductive environment of the colon, have been utilised as reduction-responsive carriers [16,29,30]. Because sodium alginate is not susceptible to enzymatic and hydrolytic degradation in the GIT, it is an ideal material for preparing nanocarriers that target the colon [28,31]. Lack of specificity in early colonic drug delivery systems could result in large fluctuations in drug levels release into the colon [32]. More specific systems can be achieved by conjugating carriers with wheat germ agglutinin (WGA) [33,34], which can bind specifically to the cell membrane of colon cells [34,35].

The conjugation of 3-mercaptopropionic acid with sodium alginate as thiolated nanocarrier has not been well established thus far. The tagging of targeting ligand, WGA with carboxyl groups at the backbone of sodium alginate bind specifically to the N-acetylglucosamine and sialic acid on the membrane of the cancer cells. This targeted drug delivery system provides prolonged circulation time, enhanced cellular-uptake, higher drug accumulation at tumour site, avoiding lysosomal degradation and specific release of drug at targeted site.

Therefore, in this study, fWGA-conjugated nanoparticles derived from thiolated sodium alginate were synthesised to improve the delivery of DTX to colon cancer cells. The first step was binding the thiol groups to sodium alginate. Nanoparticles then were prepared using a self-assembly technique in which thiol groups were air-oxidised to form disulfide linkages in the polymer matrix. The nanoparticles were further conjugated with fWGA and characterised by TEM, SEM, and DLS. The stability, pH sensitivity, reduction response, in vitro release of DTX, cell viability, and uptake of nanoparticles by cells were also investigated.

## 2. Materials and Methods

### 2.1. Materials

Sodium alginate, 1-(3-dimethylaminopropyl)-3-ethylcarbodiimide hydrochloride (EDAC), 3-mercaptopropionic acid, ≥99%, glycine, *N*-hydroxysuccinimide (NHS), dodecyl sulfate sodium salt, sodium nitroprusside, 5,5′-dithiobis(2-nitrobenzoic acid) (Ellman’s reagent), and potassium bromide were purchased from Sigma Aldrich (Saint Louis, MO, USA). GSH was acquired from Calbiochem (Tokyo, Japan). Disodium hydrogen phosphate (Na_2_HPO_4_·2H_2_O), potassium dihydrogen phosphate (KH_2_PO_4_), sodium hydroxide (NaOH), and ammonium hydrogen carbonate were purchased from R&M Chemicals (Essex, UK). DTX was obtained from TCI (Tokyo, Japan). The micro bicinchoninic acid (BCA) protein assay kit was acquired from Thermo Fisher (San Diego, CA, USA). fWGA was purchased from Vector Laboratories (Burlingame, CA, USA). 3-(4,5-Dimethylthiazol-2-yl)-2,5-diphenyltetrazolium bromide (MTT) and Tween 20 were obtained from Sigma Aldrich. Roswell Park Memorial Institute 1640 (RPMI), penicillin-streptomycin, foetal bovine serum (FBS) and paraformaldehyde (PFA) were acquired from Nacalai Tesque (Kyoto, Japan). Dulbecco’s modified eagle medium (DMEM), trypsin-ethylenediaminetetraacetic acid, and trypan blue were purchased from Gibco (Grand Island, NY, USA). Phosphate buffered saline (PBS), Triton X-100, and bovine serum albumin (BSA) were obtained from Invitrogen (Carlsbad, CA, USA). Anti-clathrin antibody (primary antibody) and goat anti-rabbit IgG H&L (Alexa Fluor^®^ 647) (secondary antibody) were acquired from Abcam (Cambridge, MA, USA). ProLong diamond antifade mountant with 4′,6-diamidino-2-phenyllindole (DAPI) was purchased from Thermo Fisher (Waltham, MA, USA).

### 2.2. Synthesis and Characterisation of Thiolated Sodium Alginate

Thiolated sodium alginate was synthesised according to previous reported method with some modifications [36]. Briefly, 1 mole of sodium alginate (2.16 g) was added to 50 mL of deionised water and stirred and heated until completely dissolved. Then, 1 mole each of 3-mercaptopropionic acid (1.29 mL) and hydrochloric acid (2.00 mL, 7 N) were added dropwise into the solution. This mixture was stirred at 80 °C for 150 min under reflux conditions. The mixture was then added into ethanol (100 mL) under flowing nitrogen. The white precipitate of thiolated polymer (MPA1) acquired from the reaction was washed twice with ethanol. The resulting precipitate was kept at −80 °C for 4 h and lyophilised using a freeze dryer for 24 h. Synthesis of thiolated polymers for different feed molar ratios of sodium alginate with 3-mercaptopropionic acid (1:2, 1:3, and 1:5) was carried out according to the procedures stated above, and the polymers were labelled as MPA2, MPA3, and MPA5.

The presence of the thiol groups in MPA samples was confirmed using sodium nitroprusside reagent [16]. Ellman’s method is a spectrophotometric method which is based on the reaction of 5,5′-dithiobis-(2-nitrobenzoic) acid (DTNB) with SH groups to produce yellow chromophore with maximum absorbance at 412 nm [37,38]. Therefore, Ellman’s method was utilised to determine the thiol contents of the samples. Sodium alginate (negative control) and MPA samples were subjected to Fourier transform infrared spectroscopy (FTIR) and ^1^H-nuclear magnetic resonance (NMR) characterisations.

### 2.3. Preparation and Characterisation of Blank Disulfide Cross-Linked Nanoparticles

Blank disulfide cross-linked nanoparticles were prepared following a modified previously reported method [20]. Briefly, 10 mg of the thiolated sodium alginate was dissolved in 10 mL of 0.25 N NaOH aqueous and stirred for 1 h in an ice bath. The solution then was left to react in the dark at 25 °C for 24 h. The solution was ultrasonicated for 15 min and cooled down to room temperature. The resulting solution was stored at −80 °C for 4 h and lyophilised for 24 h. The nanoparticles formulated using MPA1, MPA2, MPA3, and MPA5 were named MP1, MP2, MP3, and MP5, respectively. The presence of the thiol groups in the nanoparticles was examined, and their concentrations were determined. The zeta potential and mean diameter of the nanoparticles were examined using the DLS method. The surface morphology of the nanoparticles was studied using SEM and TEM.

#### 2.3.1. Stability Studies of Nanoparticles

The nanoparticles were stored in buffer solution (pH 7.4) at 25 °C for 30 d. The mean diameter and zeta potential of the nanoparticles before and after 30 d were evaluated to determine the long-term stability of the nanoparticles [39].

#### 2.3.2. pH Sensitivity Studies of Nanoparticles

The aqueous nanoparticle suspensions were separated into seven parts. Each part’s pH was adjusted to pH 2–8 using 0.1 M NaOH and 0.1 M HCl. After 24 h, the mean diameter of the nanoparticles for each pH suspension was measured at 25 °C to examine the response of the nanoparticles towards the pH changes, which mimicking the microenvironment of the human GIT [20].

#### 2.3.3. Reduction Response Studies of Nanoparticles

The nanoparticles were placed in buffer solution (pH 7.4) and separated into three parts. The first part was used as the control, whereas the second and third parts contained added GSH at 10 µM and 10 mM, respectively. The mean diameter and zeta potential of the nanoparticles were determined after 24 h to assess the redox behaviour of the nanoparticles, simulating the reductive microenvironment of the cancer cells [40].

### 2.4. Preparation, Characterisation and In Vitro Drug Release Studies of Drug-Loaded Nanoparticles

To prepare the DTX-loaded nanoparticles, 10 mg of the thiolated sodium alginate was dissolved in 10 mL of 0.25 N NaOH aqueous under stirring. Then, 0.01% *w*/*v* of ethanolic DTX solution was added to the polymer solution and stirred at 4 °C for 1 h. The solution was left to react in the dark at 25 °C for 24 h and then ultrasonicated for 15 min [20]. The drug-loaded nanoparticles were recovered by centrifugation at 12,000 rpm for 30 min and washed several times with ethanol to remove the free drug. The nanoparticles then were lyophilised to yield drug-loaded nanoparticles. This procedure was applied to MPA1, MPA2, MPA3, and MPA5 polymers. The DTX-loaded sodium alginate nanoparticles were named DMP1, DMP2, DMP3, and DMP5, respectively.

The UV absorbance of the recovered supernatant that containing the unbound DTX were determined at 230 nm using a UV-Vis spectrophotometer (Cary 50, Varian, Melbourne, VIC, Australia) [41]. The concentration of DTX in DMP1–5 were evaluated using calibration curve constructed from DTX standard solutions in ethanol (R^2^ = 0.9995) (Figure S1). The loading efficiency for triplicate samples was calculated, and the results were averaged using the following formula:

$$\mathrm{Loading}\;\mathrm{efficiency}\;(\%)\;=\;\frac{\mathrm{Wt}\;-\;\mathrm{Wf}}{\mathrm{Wt}}\;\times \;100$$

where Wt is the initial amount of DTX used for drug loading and Wf is the total amount of DTX recovered in the supernatant. The DMP samples were analysed by DLS using a zetasizer (Nano ZS, Malvern Instruments, Worcestershire, UK) to evaluate the mean diameter and zeta potential of the nanoparticles. SEM and TEM were used to analyse the morphology of the nanoparticles [42,43].

DMP2 was chosen for the drug release study based on the results of a loading efficiency analysis. In vitro drug release studies were conducted at 37 ± 0.1 °C in a shaker incubator at 50 rpm. Hydrochloric acid solution (pH 1.0, stomach), phosphate buffer solution (pH 7.4, small intestine), and phosphate buffer solution (pH 6.0) with and without GSH (colon) were used to simulate the pH and reducing environment of the GIT [20,44].

Drug-loaded samples (20 mg) were divided and placed into two dialysis bags and suspended in conical flasks containing 10 mL of simulated stomach solution for 2.0 h. The samples were then placed in the simulated small intestine solution for 3.0 h. Next, the samples were divided into two parts: one was placed in phosphate buffer solution (pH 6.0) as the control, and the other was placed in phosphate buffer solution (pH 6.0) containing 25 mM GSH. At predetermined time intervals, 250 µL of the release medium was removed and replaced with an equal volume of fresh medium (sink conditions). The concentration of DTX was measured at 230 nm absorbance using a UV-Vis spectrophotometer [45]. The calibration curves at pH 1.0 (R^2^ = 0.9966), 7.4 (R^2^ = 0.9969) and 6.0 (R^2^ = 0.9977) buffer media were constructed using standard DTX solutions to evaluate the amount of DTX released at predetermined pH and time (Figure S2). The cumulative drug release for triplicate samples was determined, and the results were averaged using the following formula:

$$\mathrm{Cumulative}\;\mathrm{drug}\;\mathrm{release}\;(\%)\;=\;\frac{\mathrm{Amount}\;\mathrm{of}\;\mathrm{drug}\;\mathrm{released}\;\mathrm{at}\;\mathrm{predetermined}\;\mathrm{time}}{\mathrm{Initial}\;\mathrm{amount}\;\mathrm{of}\;\mathrm{drug}\;\mathrm{in}\;\mathrm{nanoparticles}}\;\times \;100$$

### 2.5. Preparation and Characterisation of fWGA-Conjugated Blank Nanoparticles

Nanoparticles were conjugated with fWGA [46]. MP2 was chosen for use in this procedure due to its promising characteristics (small particle size and high stability). A nanoparticle sample (13 mg) was dissolved in 1 mL of PBS. The nanoparticle suspension was mixed with 1 mL of 3.5% *w*/*v* EDAC and 1 mL of 0.15% *w*/*v* NHS. The mixture was incubated at 25 °C for 2 h followed by centrifugation at 12,000 rpm to remove the supernatant. The obtained nanoparticles were washed three times with ethanol, resuspended in 1 mL of PBS, and mixed with 200 µL of 0.1% *w*/*v* fWGA. The mixture was left to react at room temperature for 18 h. Next, 200 µL of 20% *w*/*v* glycine in PBS was pipetted into the solution, and the mixture was left to react for 1 h. The nanoparticles were washed three times with ethanol and then lyophilised using a freeze dryer for 24 h. The products obtained were labelled as fMP2.

The fWGA conjugation efficiency of the nanoparticles was determined by measuring the absorbance at 562 nm using a UV/Vis microplate reader (ELx808 Absorbance Reader, Biotek, VT, USA) according to previously reported method [47]. The conjugation efficiency for triplicate samples was determined, and the results were averaged using the following formula:

$$\mathrm{Conjugation}\;\mathrm{efficiency}\;=\;\frac{\mathrm{Amount}\;\mathrm{of}\;\mathrm{fWGA}\;\mathrm{conjugated}\;\mathrm{on}\;\mathrm{the}\;\mathrm{nanoparticles}}{\mathrm{Weight}\;\mathrm{of}\;\mathrm{lyophilised}\;\mathrm{nanoparticles}}\;\times \;100$$

The mean diameter and zeta potential of fMP2 samples were analysed using the DLS method. Moreover, the stability, pH sensitivity, and reduction response profiles of the nanoparticles were investigated using the DLS method.

### 2.6. Preparation and Characterisation of fWGA-Conjugated Drug-Loaded Nanoparticles

DMP2 had the highest drug loading efficiency of the DMPs tested, and it was used for further studies. The conjugated nanoparticles were labelled as fDMP2. The fWGA conjugation efficiency of the nanoparticles was evaluated using the micro BCA protein assay kit. The mean diameter and zeta potential of the nanoparticles were determined using the DLS method. The morphology of the nanoparticles was studied using SEM and TEM. The stability and drug release profiles of the nanoparticles were also investigated using the DLS and in vitro dialysis methods, respectively.

### 2.7. Cytocompatibility Studies—MTT Assay

HT-29 and L929 cells were cultured in RPMI and DMEM (10% FBS; 1% penicillin-streptomycin), respectively [48]. Cells at a density of 1 × 10^4^ cells per well were seeded in a 96-well plate and incubated in a 5% CO_2_, 95% air humidified incubator at 37 °C. Nanoparticles were dissolved in growth medium and serially diluted to prepare different treatment concentrations. After 24 h of incubation, the medium was withdrawn from the wells and replenished with 100 µL of the nanoparticle suspensions. The treated cells were further incubated at 37 °C. Untreated cells in medium served as the negative control. After incubation for 24 h, the medium was withdrawn from the wells and replenished with 100 µL of fresh medium. Next, 10 µL of 5 mg/mL MTT solution was pipetted into the wells. After incubation for 4 h, the MTT solution was withdrawn from the wells and replenished with 100 µL of dimethyl sulfoxide (DMSO). The plate was shaken prior to measurement. The optical density of the solution was determined using a UV/Vis microplate reader at 570 nm [49]. Cell viability for triplicate samples was calculated as follows, and the results were averaged:

$$\mathrm{Cell}\;\mathrm{viability}\;(\%)\;=\;\frac{\mathrm{Absorbance}\;\mathrm{of}\;\mathrm{cells}\;\mathrm{treated}\;\mathrm{with}\;\mathrm{sample}}{\mathrm{Absorbance}\;\mathrm{of}\;\mathrm{untreated}\;\mathrm{cells}}\;\times \;100$$

### 2.8. Cellular Uptake Studies

#### 2.8.1. Cellular Uptake Efficiency

To assay cellular uptake of nanoparticles quantitatively, HT-29 cells at a density of 1 × 10^4^ cells per well were seeded in a 96-well microplate in a 5% CO_2_, 95% air humidified incubator at 37 °C. After incubation for 24 h, the medium was withdrawn. The cells were washed three times with pre-warmed PBS, and then 100 µL of the DTX-loaded nanoparticles in PBS (concentration of 3.9–125.0 µg/mL) was pipetted into the wells. The cells were incubated at 37 °C for 0.5–4 h. At predetermined times, the supernatants of the wells were removed as samples (I_sample_). The wells that retained the supernatant served as positive controls (I_positive_) [50]. After withdrawing the supernatants, PBS was used to wash the cells three times. The cells then were solubilised with 100 µL of Triton X-100 (0.5% Triton X-100 in 0.2 N NaOH). Fluorescence intensities were measured using a microplate fluorescence reader (FLUOstar Omega, BMG LABTECH, Ortenberg, Germany). The excitation and emission wavelengths used were 485 and 520 nm, respectively. The uptake efficiency formula was calculated as follows:

$$\mathrm{Uptake}\;\mathrm{efficiency}\;(\%)\;=\;\frac{I_{\mathrm{sample}}\;-\;I_{\mathrm{negative}}}{I_{\mathrm{positive}}\;-\;I_{\mathrm{negative}}}\;\times \;100$$

where I_negative_ represents the fluorescence intensity retrieved from the wells containing the cells in PBS.

#### 2.8.2. Fluorescent Imaging

Fluorescent imaging of the cellular uptake of the nanoparticles was carried out according to a method described previously with some alterations [51]. Briefly, HT-29 cells were seeded onto a 6-well plate at 1 × 10^5^ cells per well and incubated in a 5% CO_2_, 95% air humidified incubator at 37 °C. After 24 h of incubation, the medium was withdrawn, and PBS was used to wash the wells. Next, 2 mL of 50 µg/mL Alexa Fluor fWGA-labelled nanoparticles in PBS was pipetted into the wells and incubated for 2 h. The solution was then withdrawn, and PBS was used to wash the wells. The wells containing the cells were viewed under a fluorescence microscope (Olympus CKX41, Olympus, Tokyo, Japan) to observe the cells using bright field and a blue fluorescence filter under 40 × magnification with a numerical aperture of 0.6.

#### 2.8.3. Confocal Laser Imaging

Coverslips were placed in the wells of a 6-well plate. HT-29 cells were seeded onto the coverslips at a density of 1 × 10^5^ cells per wells and incubated at 37 °C in a 5% CO_2_, 95% air humidified incubator. After 24 h of incubation, the medium was withdrawn, and pre-warmed PBS was used to wash the wells. Next, 2 mL of 50 µg/mL nanoparticles in PBS was added in the wells and incubated for 2 h. The solution was withdrawn, and ice-cold PBS was used to wash the wells twice. Next, 2 mL of 4% PFA was used to fix the cells for 15 min, and ice-cold PBS was used to wash the cells twice [52]. Then, 2 mL of Triton X-100 (0.1% in PBS) was added to the cells and incubated for 15 min on ice. PBS was used to wash the cells three times. Next, 2 mL of blocking buffer (PBS containing 1% BSA and 0.2% Tween-20) was added to the cells, which then were incubated for 1 h. Primary and secondary antibodies were diluted at 1:200 with blocking buffer. The diluted primary antibody (anticlathrin antibody) was pipetted into the wells and incubated at 4 °C in a dark humidified chamber for 24 h. PBS was used to wash the cells five times. Subsequently, the diluted secondary antibody [goat antirabbit IgG H&L (Alexa Fluor^®^ 647)] was pipetted into the wells followed by 1 h of incubation in the dark at 4 °C. PBS was used to wash the cells five times. ProLong diamond antifade mountant with DAPI was used to mount the coverslips onto glass slides, and the slides were viewed under a confocal microscope (Zeiss CLSM 710, Zeiss, Jena, Germany) using an oil-immersion objective with 63 × magnification and a numerical aperture of 1.4 to observe the fluorescence emitted by the cells using DAPI, FITC, and A647 filters.

### 2.9. Statistical Analysis

The data were expressed as the mean ± standard deviation (SD). Error bars indicate standard deviation from the mean (*n* = 3). The paired-sample *t* test was applied to analyse the data from the thiol content and stability nanoparticle studies. Dunnett test is a *t*-statistic used to compare each treatment group mean with the mean of the control group [53]. Dunnett’s (two-sided) *post hoc* test was applied to analyse the results of the reduction response and in vitro drug release studies. One-way analysis of variance with Tukey’s HSD (honestly significant difference) *post hoc* test was applied to analyse data from the in vitro nanoparticle cytotoxicity studies for paired comparisons of mean values. *p <* 0.05 was regarded as indicative of statistical significance.

## 3. Results and Discussion

### 3.1. Characterisation of MPA1–MPA5

**Scheme 1:**
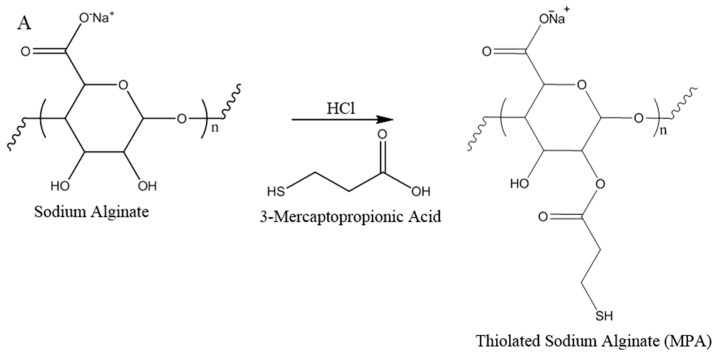

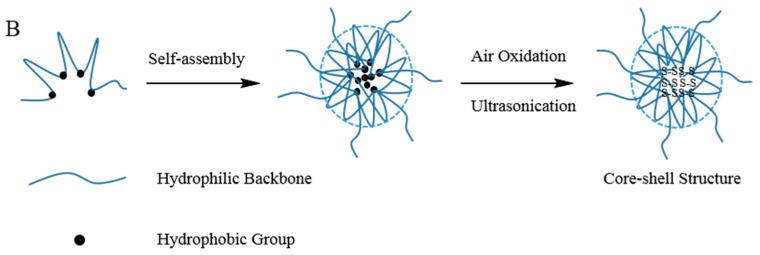
Synthetic scheme of (**A**) MPA and (**B**) assembly process of disulfide cross-linked nanoparticles.

Synthesis of MPA polymers involved esterification of the hydroxyl groups of sodium alginate with the carboxyl group of 3-mercaptopropionic acid in the presence of hydrochloric acid (Scheme 1A) [36,54].

Due to the acidic conditions, sodium alginate was transformed into insoluble alginic acid, but it could be neutralised with NaOH to form water-soluble thiolated sodium alginate [55]. The average yield of the products was 73.4%. Thiol-acids are soluble in ethanol; thus, repeated washing of the products with ethanol was sufficient to remove the unreacted thiol-acids [56]. The product obtained was white in colour and dissolved readily in alkaline water [36]. The sodium nitroprusside test of the polymers showed positive results, as the colour changed from yellow to reddish purple. The thiol contents of MPA1, MPA2, MPA3, and MPA5 were 1.49, 2.43, 2.64, and 3.16 mM/g polymer, respectively. MPA5 had the highest thiol content because the highest number of moles of 3-mercaptopropionic acid was used in the reactions. This result suggests that increasing concentrations of thiol-acids triggered the reactions required to produce polymers with higher thiol group conjugation efficacy [20].

**Figure 1:**
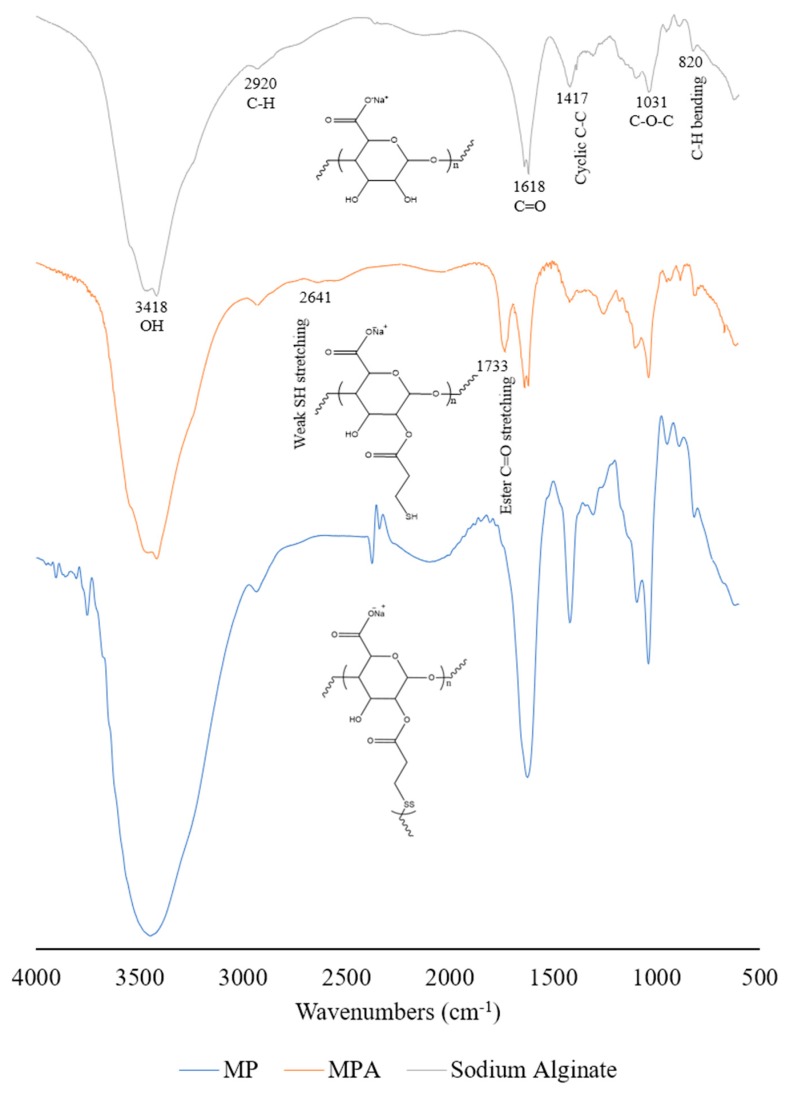
FTIR spectra of sodium alginate, MPA and MP.

The FTIR spectra of sodium alginate and MPA were used to identify their respective functional groups (Figure 1). The spectrum of sodium alginate revealed a broad absorption band of OH stretching at 3418 cm^−1^ and CH stretching at 2920 cm^−1^ [36]. Peaks appearing at 1618 cm^−1^ and 1417 cm^−1^ were assigned to symmetric and asymmetric stretching vibrations of carboxylate anions, respectively [57]. The adsorption bands at 1031 cm^−1^ and 820 cm^−1^ were attributed to COC cyclic ether stretching and cyclic CH bending [58]. In comparison to sodium alginate, the spectrum of MPA showed a new but relatively weak characteristic peak at approximately 2641 cm^−1^; this outcome was attributed to SH stretching of the thiol group [56]. An additional peak at 1733 cm^−1^ was attributed to ester carbonyl group stretching [36]. The FTIR results with the presence of additional peaks of ester and SH stretching suggest the conjugation of thiol to sodium alginate was successful. The absence of SH peak in MP spectrum indicates the oxidation of thiols to form disulfide bonds.

**Figure 2:**
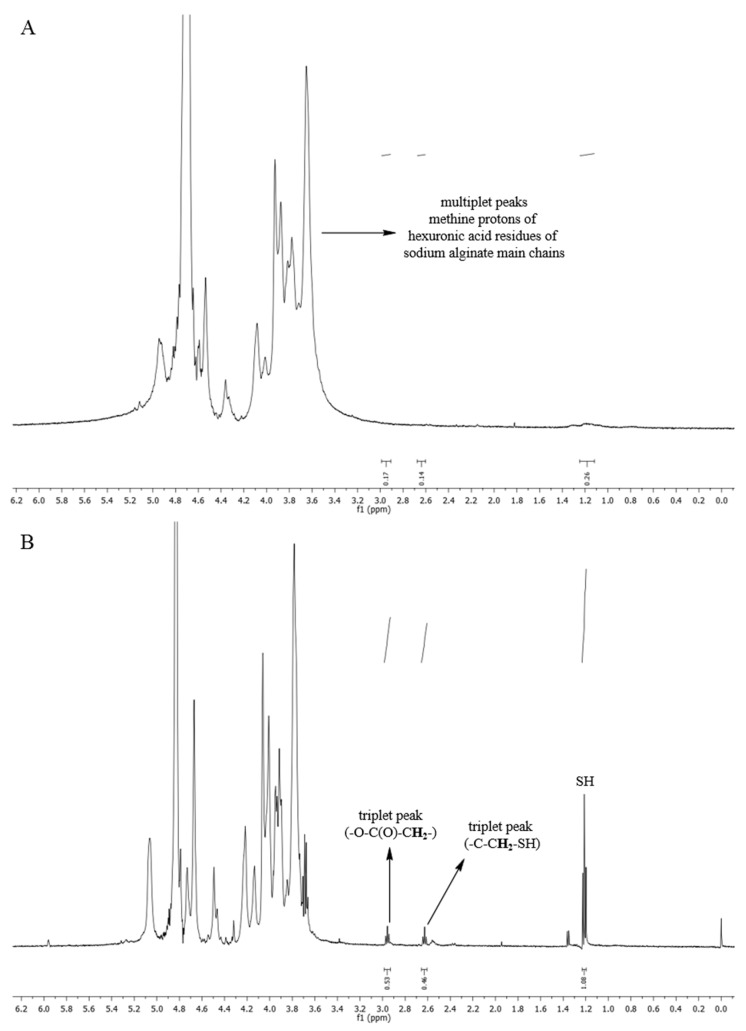
^1^H-NMR spectra of (**A**) sodium alginate and (**B**) MPA.

^1^H-NMR analysis was carried out to further confirm the conjugation of thiol to sodium alginate (Figure 2). The multiplet peaks between 3.7 and 5.0 ppm were assigned to the protons of methine groups of hexuronic acid moieties from the sodium alginate backbone [59]. The MPA spectrum showed typical peaks of the protons from the sodium alginate chain. New peaks and increased peaks present in the MPA spectrum indicated the emergence of new functional groups bonded to the alginate chain. The additional triplet peaks at 2.6 ppm (-O-C(O)-C**H_2_**-) and 2.9 ppm (-C-C**H_2_**-SH) in the MPA spectrum were attributed to the proton in 3-mercaptopropionic acid [54]. Furthermore, the presence of additional peaks at 1.2 ppm in the MPA spectrum was due to the chemical shift of SH [40]. The SH grafting rate was calculated to be 75.9% based on the subtraction of the integral peak area (1.2 ppm) in the MPA and sodium alginate spectra.

### 3.2. Characterisation of MP1–MP5

**Figure 3:**
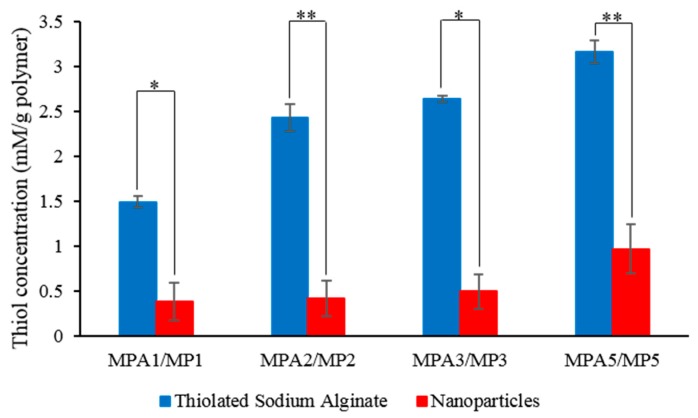
Comparison of the concentrations of thiol between MPA polymers and MP nanoparticles (mean ± SD, *n* = 3); ** (*p* < 0.01) and * (*p* < 0.05) indicate significant differences between the samples.

Disulfide cross-linked nanoparticles were formed via self-assembly, whereby the adjacent thiol groups in the nanoparticle core were cross-linked via air oxidation under ultrasonication (Scheme 1B) [20]. The sodium nitroprusside test of the polymers showed the absence of thiol, as the solution remained yellow in colour. This indicates that disulfide bonds were successfully formed from the air oxidation of the thiols [16]. The sodium nitroprusside reagent could not enter into the nanoparticle core to react with disulfide bonds. The thiol contents in MPA polymers (Figure 3) decreased after formulation into nanoparticles, whereas all MP nanoparticles showed significant differences in thiol contents from MPA polymers. The thiol contents varied from MPA1 to MPA3 because the thiol groups readily reacted with Ellman’s reagent. However, there were no significant differences between the thiol concentrations of MPA1 to MPA3 determined using Ellman’s method because the Ellman’s reagent could not react with the compact structure of the disulfide bonds. The decrease in thiol contents was in the following order: MP2 (82.7%) > MP3 (81.1%) > MP1 (73.8%) > MP5 (69.3%). MP2 exhibited the most promising rate of decrease in thiol contents (*p* < 0.01) among all nanoparticles tested; thus, it was chosen for further characterisation. The remaining thiol content in MP2 was 17.3% of the initial value. This large decrease in thiol content in the nanoparticles indicates that most thiol groups were air-oxidised to develop disulfide bonds [20,39].

### 3.3. Characterisation of DMP1–DMP5

DMP2 had the highest loading efficiency (17.8 ± 2.4%), followed by DMP3 (12.7 ± 3.4%), DMP5 (11.2 ± 2.7%), and DMP1 (10.0 ± 6.6%) (mean ± SD, *n* = 3). The low loading efficiency maybe due to the cohesive interactions among the polymer chains that cause limited space to retain the DTX in the polymer matrix [60]. Also, DTX might leak from the inner core during sonication. The amount of disulfide linkages formed in DMP2 was the highest amongst all the formulations, and thus the loading efficiency of DMP2 was the highest. This is due to the stronger hydrophobic interactions between the disulfide bonds and the DTX. Therefore, DMP2 was chosen for use in subsequent experiments. DTX was successfully loaded into the nanoparticles during the self-assembly process by mixing the ethanolic DTX solution with the aqueous thiolated sodium alginate solution. During the mixing process, a precipitate of DTX-loaded nanoparticles was formed due to the interfacial turbulence formed between the two immiscible liquid phases [61]. DTX was loaded into the nanoparticles via hydrophobic interactions formed between disulfide bonds and DTX within the nanoparticles, and these interactions enhanced the stability of the DTX-loaded nanoparticles. Furthermore, the solubility of the hydrophobic drug was improved by loading the drug into the nanoparticles [40].

### 3.4. Characterisation of fMP2 and fDMP2

In the two-step carbodiimide process, amide bonds were formed by conjugation of the fWGA primary amino groups to the sodium alginate carboxyl groups [62]. The Micro BCA protein assay kit was used to examine the fWGA concentration because the existence of amino acids, such as tryptophan, cysteine, tyrosine, and cystine, as well as peptide bonds in the fWGA is responsible for the reduction of Cu^2+^ ions in the BCA [63]. Purple fluorescent exhibited by the BCA-Cu+ complex was then determined at 562 nm according to the protocol provided by the Micro BCA protein assay kit. The amount of fWGA conjugated to fMP2 nanoparticles was 19.1 ± 4.4 µg fWGA/mg nanoparticles. The fWGA conjugation efficiency for the fMP2 nanoparticles was 16.7%. fWGA was further conjugated to DMP2 to form fDMP2, which contained 16.1 ± 2.1 µg fWGA/mg nanoparticles. fDMP2 had a conjugation efficiency of 14.1%. This fWGA conjugation to the nanoparticles was governed by particle size, whereby small MP2 and DMP2 nanoparticles had a high specific surface area for fWGA conjugation [64].

### 3.5. DLS Analysis

The MD, PDI, and zeta potential of MP1–5 nanoparticles were determined using the DLS method (Table 1). Most of the MD values for nanoparticles were ~200 nm, with PDI values below 0.2. These results suggest that MP nanoparticles have a narrow size distribution due to the high zeta potential in MP nanoparticles, reflecting stronger repulsive forces between nanoparticles that result in higher stability and more uniform size distributions [65]. The zeta potentials of MP2 nanoparticles were greater than −40 mV, indicating high stability of negatively charged nanoparticles from the anionic sodium alginate in the nanoparticle composition [66]. It was reported that these negatively charged nanoparticles showed high cellular uptake efficacy due to the electrostatic interactions with cellular membranes, and they easily adhered to inflamed tissues of the colon, which secretes positively charged proteins in situ, such as eosinophil and bactericidal proteins [20].

An increase in MD and PDI values was observed after loading DTX into the nanoparticles, indicating a successful loading process. For DMP2, the MD increased from 204 nm (MP2) to 219 nm. In comparison to MP, DMP nanoparticles showed reduced zeta potential. The loading of hydrophobic DTX into the nanoparticle cores (hydrophobic disulfide linkages) increased the hydrophobic interactions among the nanoparticles. The repulsive forces between the nanoparticles decreased, thus resulting in lower zeta potentials [67]. There were obvious differences between the PDI and zeta potential values of MP1–5. However, the PDI and zeta potential values of DMP1–5 were very close. This was due to the lack of significantly different DTX loading efficiencies for DMP1–5.

In comparison to the MD of MP2, the MD of fMP2 increased by 70 nm to 274 nm (Table 2). The PDI values for fMP2 were below 0.3, indicating that fMP2 had a relatively uniform and narrow size distribution. The zeta potentials of fMP2 decreased due to the conjugation of positively charged fWGA to the carboxyl groups of sodium alginate chains of the nanoparticles [47]. Compared to fMP2, the MD of the fDMP2 increased by 15 nm to 289 nm. This insignificant variation of MD is probably due to the low drug loading efficiency. Despite this, fDMP2 had cancer cells targeting properties from WGA that require low dose of DTX to induce cytotoxic to colon cancer cells. The ideal particle size is <500 nm, which is the maximum size for nanoparticles to bypass the cell membrane [68]. fDMP2 had a narrow size distribution, with PDI values less than 0.4, indicating a relatively homogenous population. The zeta potential of fDMP2 decreased due to the hydrophobic interactions between the hydrophobic DTX and the disulfide linkages in the nanoparticle cores, leading to the formation of aggregation in the nanoparticles [67]. The decrease in zeta potential was also attributed to conjugation of the positively charged fWGA to the polymer chain segments [47].

### 3.6. Surface Morphology

**Figure 4:**
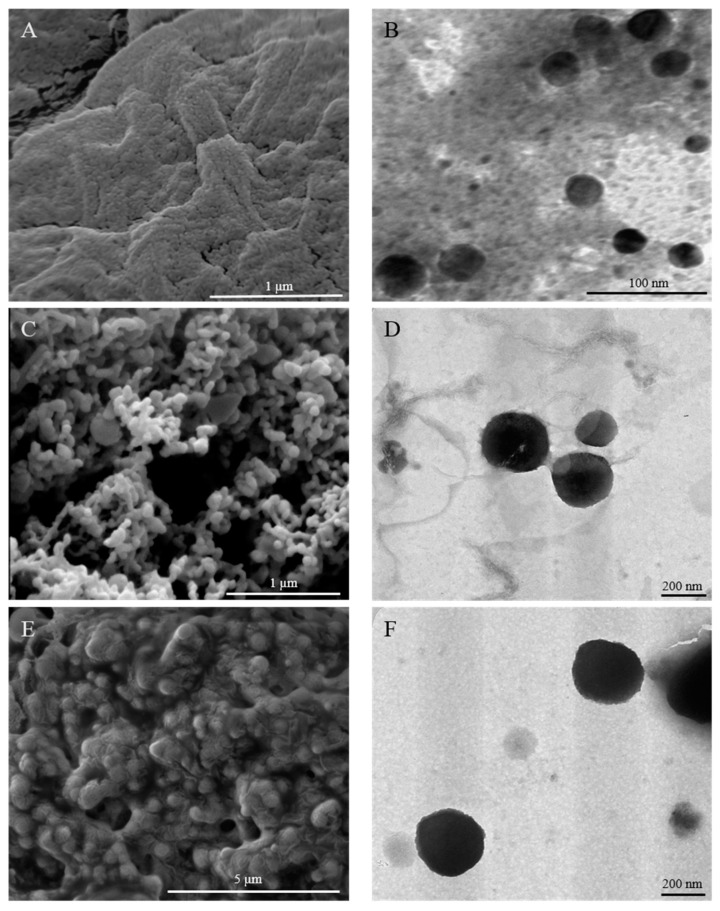
SEM micrographs of (**A**) MP2, (**C**) DMP2, (**E**) fDMP2; and TEM micrographs of (**B**) MP2, (**D**) DMP2, (**F**) fDMP2.

MP2 consisted of aggregates of spherically shaped nanoparticles with a small average size of 19 nm (Figure 4A). These aggregates formed because of the tiny size of polysaccharide-based nanoparticles, which tend to shrink and lose shape due to SEM electron bombardment [68]. TEM micrographs of MP2 nanoparticles showed spherical core-shell configurations with an average size of 52 nm (Figure 4B). The nanoparticles were well distributed, with relatively uniform size and no aggregation. This result suggests that MP2 nanoparticles were stable and well dispersed in aqueous solution.

DMP2 was spherical in shape with some tendency for agglomeration (Figure 4C). The appearance of agglomerated nanoparticles is related to the decrease in the zeta potential of the nanoparticles. The hydrophobic interactions enhanced by the loading of hydrophobic drugs into the hydrophobic nanoparticle cores led to increased attractive forces among the nanoparticles to form agglomerations [69]. Compared to the SEM micrograph of MP2, the average particle size increased from 19 nm to 128 nm for DMP2. DMP2 nanoparticles were spherical in shape and well dispersed in aqueous solution, as illustrated in TEM micrographs (Figure 4D). Drug-loaded nanoparticles with different sizes were observed. This was in accordance with the increasing values of MD. This result further confirms the successful loading of DTX into the nanoparticles. Compared to MP2 TEM micrographs, the average particle size of DMP2 nanoparticles increased from 52 nm to 237 nm.

Both SEM and TEM micrographs of fDMP2 showed nanoscale particles with spherical shapes. Agglomerations of nanoparticles in fDMP2 were observed (Figure 4E) and were attributed to low zeta potential. In comparison with the SEM micrographs of DMP2, the average particle size of fDMP2 increased by 249 nm to 377 nm. The TEM micrographs showed that fDMP2 nanoparticles were well dispersed in the aqueous solution (Figure 4F). TEM micrographs also showed that the average particle size of fDMP2 was larger than that of DMP2, increasing from 237 nm to 313 nm. These results suggest that fWGA successfully conjugated to the nanoparticles.

### 3.7. Stability Study

The stability study was conducted to examine the stability of MP nanoparticles stored in buffer solution for 30 d. Paired *t* test results showed no significant differences (*p* > 0.05) in MD after storage for 30 d in buffer solution, although most MD and PDI values of the MP nanoparticles changed slightly over time. MP2 nanoparticles showed increase of 27 nm and 0.06 in size and PDI, respectively (Table 3A). These results indicate that MP2 nanoparticles are highly stable.

No significant changes in MD (*p* > 0.05) were observed for fWGA-conjugated nanoparticles over time (Table 3B), although after 30 d of storage, the MD values of fMP2 and fDMP2 increased from 272 nm to 273 nm and 289 nm to 303 nm, respectively. PDI values of both nanoparticle types showed a small decrease of 0.01. These results indicated the high stability of fMP2 and fDMP2, which was due to the disulfide bonds formed between the inter- and intramolecular chains [20].

The inter- and intramolecular disulfide bonds formed in solution during storage play a crucial role in stabilising nanoparticles [20]. This phenomenon occurs because the cohesiveness of the polymer matrix system is improved by the development of inter- and intrachain disulfide bonds. This process results in improved swelling and dissolution behaviour of the polymer matrix system for targeted drug release [70]. Therefore, good stability of nanoparticles is desired to avoid release of the drug before it reaches the targeted site [20,39].

### 3.8. pH Sensitivity Studies

**Figure 5:**
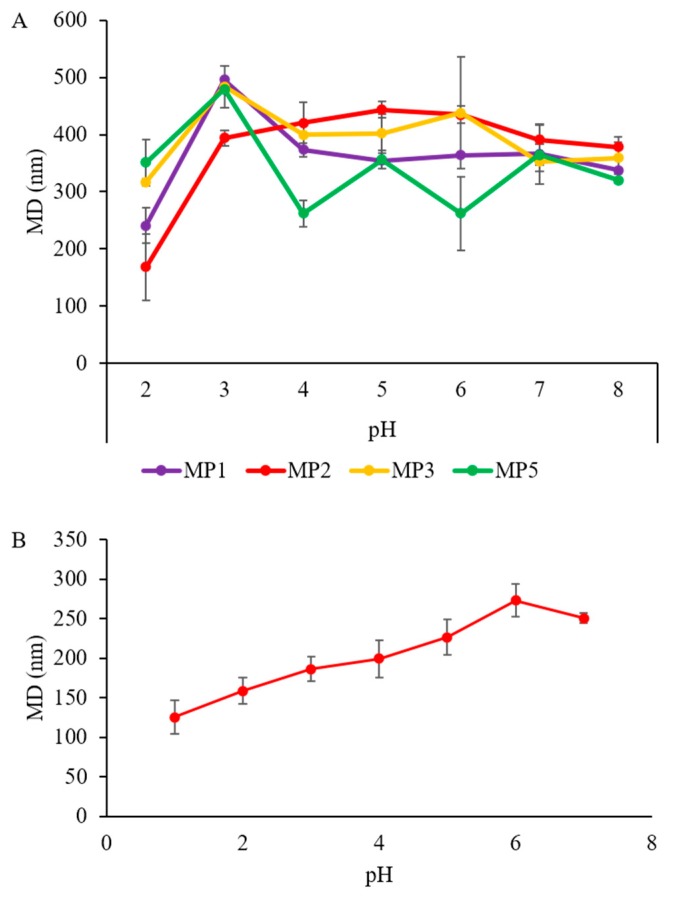
pH sensitivity profiles of (**A**) MP1–5 and (**B**) fMP2 (mean ± SD, *n* = 3).

pH sensitivity studies were conducted to determine the effects of pH on particle sizes of MPs (Figure 5A) in aqueous medium with pH values ranging from 2 to 8. MP2 showed the best results among the nanoparticles tested. The MD of MP2 nanoparticles increased steadily with increasing pH. In the range of pH 2 to 7, the MD of MP2 increased by a factor of 2.3. This result suggests that the pH sensitivity or pH-dependent swelling properties of MP2 are promising. The same trend was observed for fMP2, with maximum particle size observed at pH 7 (Figure 5B) and a steady increase in MD from pH 2 to 7. In this pH range, the MD of fMP2 increased by a factor of 1.

Particles were smaller at pH 2, possibly due to carboxyl group protonation of sodium alginate and fWGA hydrophilic segments in acidic conditions [20]. Hydrogen bonds can form between the hydrophilic backbones and cause the polymer chains to be more tightly packed, thus resulting in a smaller MD. Meanwhile, ionisation of the free carboxylic acid groups can occur at higher pH values, which can cause the breakdown of the hydrogen bonds and the formation of electrostatic repulsion among the hydrophilic segments, ultimately resulting in larger particle sizes at pH 5 to 7. However, as the pH increased to 8, a slight drop in MD was observed, possibly due to the counter ion shielding effect [71,72].

### 3.9. Reduction Response Studies

Nanoparticle size increased with increasing concentration of GSH (10 µM and 10 mM). These results suggest that the nanoparticles responded well to reductive conditions after 24 h. Although all MP nanoparticles showed good reduction responsiveness to GSH, only MP2 also had a good pH sensitivity profile (Table 4A). Thus, MP2 was chosen for further characterisation. MP2 exhibited good reductive responsiveness to the reducing environment created by GSH (reducing agent). The particle sizes of MP2 nanoparticles placed in negative control and 10 µM GSH media were not significantly different, as the MD of the MP2 nanoparticles only increased 4.9% in the 10 µM GSH treatment. This result showed that MP2 nanoparticles were stable in 10 µM GSH, which mimicked the extracellular environment, and therefore they would not release the drug before reaching the targeted site [39]. However, with the 10 mM GSH treatment, which simulated the intracellular environment of cancer cells, significant increases in MD and PDI were observed for MP (44.5%) nanoparticles relative to the negative control.

Similarly, the MD and PDI of fMP2 showed slight increases in 10 µM GSH medium relative to the negative control (Table 4B), but the difference was not statistically significant (*p* > 0.05). This result suggests that fMP2 was relatively stable in the extracellular environment (10 µM GSH). Subsequently, the MD of fMP2 increased significantly (*p* < 0.01) by a factor of 1.04 when placed in 10 mM GSH medium. This was attributed to disulfide bond cleavage in the inner core of the nanoparticles by the reducing agent GSH. Therefore, fMP2 showed great reductive responsiveness in 10 mM GSH medium, which simulated the intracellular environment of cancer cells [39]. These results demonstrated that disulfide bonds of the nanoparticles were readily cleaved by GSH, thus releasing the drug to the targeted cancer cells.

### 3.10. In Vitro Drug Release Studies

**Figure 6:**
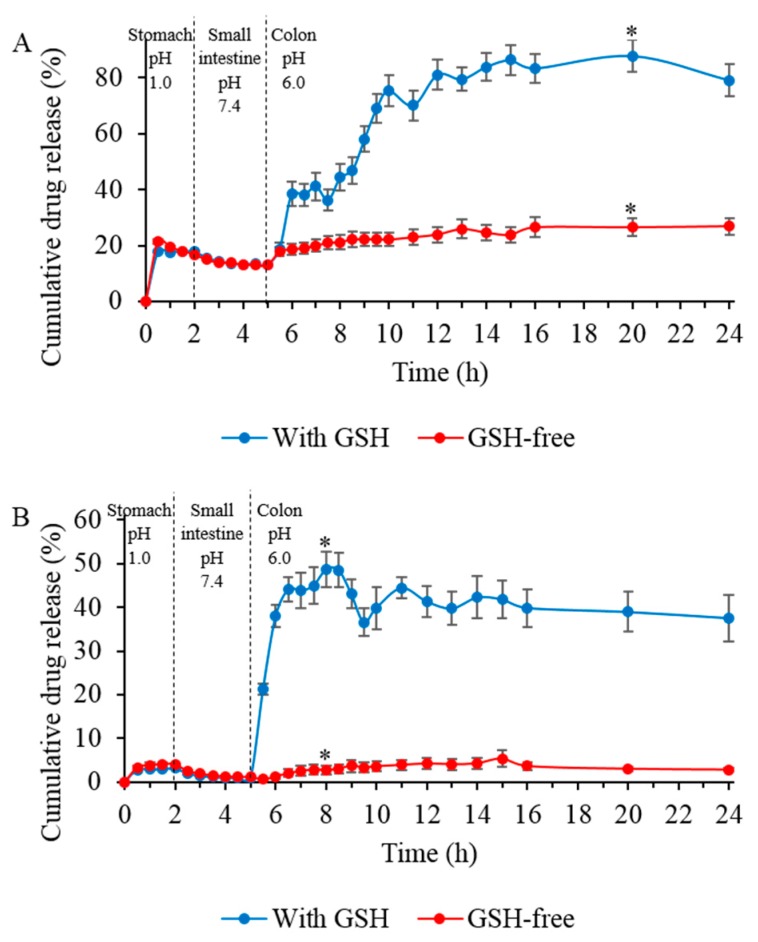
In vitro drug release profile of (**A**) DMP2 and (**B**) fDMP2 in control media (GSH-free) and in stimulated gastrointestinal media (with GSH) at predetermined pH and time (mean ± SD, *n* = 3); * (*p* < 0.05) indicates that significant differences were observed between the samples.

DTX is an anticancer agent that is extensively applied to treat various malignancies, including colon cancer [73]. The in vitro drug release profile of DMP2 in the simulated gastrointestinal media showed that initial rapid release of DTX occurred during the first 2 h in pH 1.0 medium without GSH, with 17.7% of the DTX being released (Figure 6A). Sequentially, 13.2% of DTX was released during the next 3 h in pH 7.4 medium. The highest cumulative percentage release of DTX was 87.9% in pH 6.0 GSH medium, whereas it was 26.9% in GSH-free medium (negative control). Drug release reached a plateau at 12 h.

Significant rapid initial DTX release was observed in the simulated stomach medium for DMP2, probably due to weakly bound or adsorbed free drug on the nanoparticles’ surface during the loading process [74]. The hydrophilic shell then began to swell with increasing pH, which enhanced release of the drug [20,40]. However, the release remained relatively low because DTX could not penetrate the compact network structure of nanoparticles due to the high degree of disulfide cross-linking [40]. DTX release was further enhanced when nanoparticles were placed in the reducing medium containing GSH. This caused cleavage of disulfide bonds in the inner core, thereby releasing the drug rapidly until release was nearly complete [20,39,40].

In comparison, the profile of fDMP2 revealed that 3.2% of DTX was released in the stomach medium (pH 1.0), and 0.9% was released in the small intestine medium (pH 7.4) (Figure 6B). The highest cumulative percentage drug release was 48.6% (with GSH) in the colon medium, which differed significantly (*p* < 0.05) from the 5.4% release in the negative control medium. This result suggests that DTX was released because of the reducing GSH in the colon medium. The drug release reached a plateau at 11 h.

The release data of fDMP2 were well fitted to the Higuchi model (high correlation coefficient values, *R*^2^, ≥0.83) for all of the parameters evaluated, showing that DTX is released by diffusion and dissolution mechanisms resulting from the degradation of disulfide cross-linked polymer (Table 5). In Korsmeyer-Peppas’s model, the drug release is regarded as Fickian diffusion if *n* (release exponent) is less than 0.45; non-Fickian diffusion if 0.45 < *n* < 0.89; super case-II transport (zero-order) if *n* > 0.89 [75]. Therefore, *n* is 0.64 in simulated colon condition for fDMP2, confirming that drug release follows non-Fickian diffusion and is coupled with erosion mechanisms. Also, in this case, *n* is less than 0.89, which demonstrates that the release data does not fit into zero-order model (R^2^, 0.56 in pH 1 medium). Furthermore, since the *A*/*B* values in Kopcha’s model ranged widely from 2.44 to 6.46, this further indicates that diffusion predominates over erosion resulting from pH sensitivity of the fDMP2 nanoparticles. fDMP2 showed poor linearity with respect to first-order model (R^2^, 0.58) in pH 1 medium, showing that the drug release rate is independent of concentration of drug due to the low water solubility of the DTX. Meanwhile, poor fitting in Hixson-Crowell’s model (R^2^, 0.02 in pH 1 medium) demonstrates that there is no change in the surface area and diameter of the matrix as a function of time for the release mechanism.

The highest cumulative percentage release of DTX for fDMP2 (48.6%) was lower than that of DMP2 (87.9%). No burst release was observed during the first 5 h because the conjugation of fWGA imparted polymer chain rigidity through modification of the carboxyl groups of sodium alginate [55,76]. Less chain flexibility affected the rate and threshold of swelling of the polymer, resulting in the lower rate of drug release [77]. Although conjugation of fWGA to the nanoparticles could have acted as a protective layer for the nanoparticles, it also acted as a diffusion barrier to release of the drug from the inner core of the nanoparticles. Therefore, the observed lower level of DTX cumulative release for fDMP2 was due to the increased steric hindrances formed by fWGA conjugation.

Overall, the release mechanism of DTX is based on the pH sensitivity and reductive responsiveness of the nanoparticles. In this study, the reductive environment of the colon was simulated using GSH with a standard potential that ranged from −205 to −260 mV [78]. Disulfide bonds are reversibly cleaved in the higher GSH concentration (2–10 mM) present in the intracellular reductive environment relative to the extracellular environment (2–20 µM GSH) [40]. The concentration of GSH in cancer cells is at least seven times greater than that in normal cells, inducing the reduction of disulfide bonds and thereby releasing DTX in the colon cancer cells [39]. These results indicate that controlled drug release in the GIT within a set period of time could be achieved.

Dunnett’s (two-sided) *post hoc* test results showed that the release of DTX from both DMP2 and fDMP2 in the colon medium with GSH at pH 6.0 was significantly different (*p* < 0.05) compared to that of stomach and small intestine conditions. No significant drug release was observed in the stomach and small intestine relative to the negative control. This outcome implies that GSH is crucial for cleaving the nanoparticles’ disulfide linkages and that it is required for the release of DTX in the colon. This result also suggests that both nanoparticle types exhibited targeted release of DTX to colon cancer cells.

### 3.11. Cytocompatibility Studies—MTT

**Figure 7:**
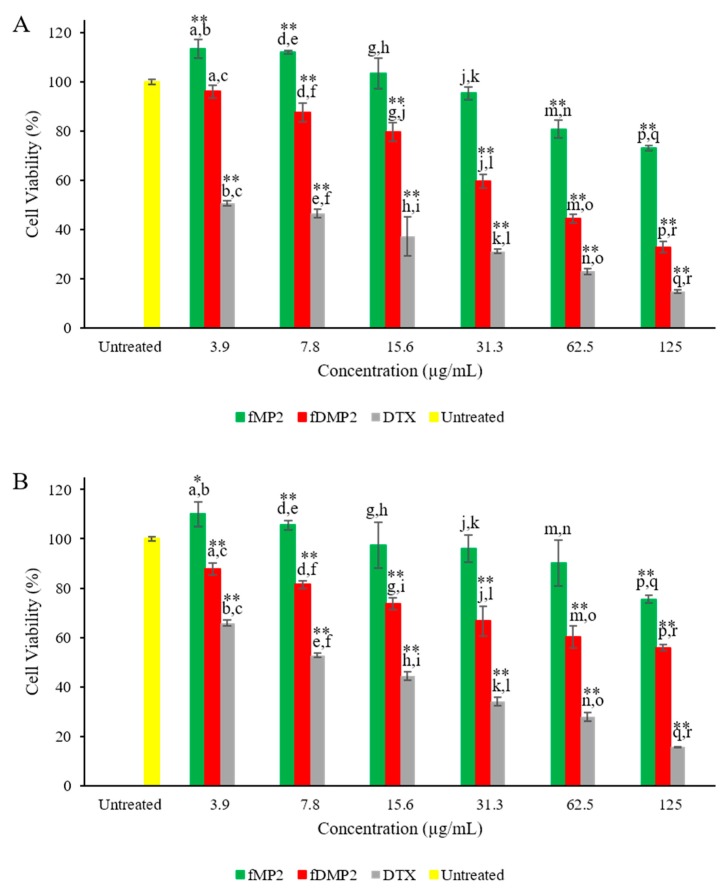
Comparison of effects of fMP2, fDMP2, and DTX on (**A**) HT-29 cells and (**B**) L929 cells (mean ± SD, *n* = 3); the samples with same alphabets a–r indicate significant difference between them, while, sample with * indicates (*p* < 0.05) compared to untreated cells.

The HT-29 cells treated with fMP2 and fDMP2 showed a dose-dependent effect. HT-29 cells remained viable (*p* > 0.05) until the concentration of fMP2 rose higher than 62.5 µg/mL (80.8%) (Figure 7A). Statistically significant differences (*p* < 0.01) were observed between the fDMP2 and fMP2 samples, indicating that DTX released from the nanoparticles was involved in lowering cell viability. Cell viability of HT-29 was much lower when cells were exposed to free DTX relative to fDMP2. This further confirmed that fDMP2 reduced the cytotoxic effect of the free drug. Although free DTX had a higher cytotoxic effect than that of fDMP2, DTX was only able to dissolve in an organic solvent such as DMSO before dilution with the culture medium. However, loading the drug into the nanoparticles enhanced the solubility of DTX because fDMP2 was readily soluble in aqueous culture medium. HT-29 cells treated with 125 µg/mL fMP2 (73.1%) and fDMP2 (32.9%) showed the lowest cell viability.

L929 cells were chosen as normal cells because this cell line model is an established and robust cytotoxicity test model recommended by the International Standard Organisation (ISO) in ISO 10993-5 standards [79]. Dose-dependent trends were observed for fMP2 and fDMP2 against L929 cells (Figure 7B). L929 cells exposed to fMP2 with concentrations lower than 62.5 µg/mL remained highly viable, as no significant differences (*p* > 0.05) were detected when they were compared to untreated cells. Statistically significant differences (*p* < 0.01) in cell viability were observed between fDMP2 and DTX for all concentrations, attributed to the cytotoxic effect of DTX. However, the viability of cells exposed to fDMP2 was significantly higher than that of cells exposed to DTX, as indicated by *p* values > 0.05. This result explains why DTX-loaded nanoparticles reduced the cytotoxic effect of free drug on healthy, normal cells.

IC_50_ is defined as the concentration of drug required to inhibit 50% of cell viability [80] and it is calculated from the curve fitting of the cell viability versus concentration of drug. The IC_50_ values were significantly lower (*p* < 0.05) in HT-29 cells (52.9 µg/mL in fDMP2) than those in L929 cells (201.6 µg/mL in fDMP2). These results illustrate the selectivity of the drug towards HT-29 over L929 cells. This effect would be beneficial when nanoparticles are utilised for delivering anticancer drugs to colon cancer cells [34].

### 3.12. Cellular Uptake of fDMP2

Cellular uptake of fDMP2 was demonstrated in HT-29 cells. Green fluorescent fWGA was conjugated to the nanoparticles to visualise the cellular internalisation of nanoparticles in the cells.

**Figure 8:**
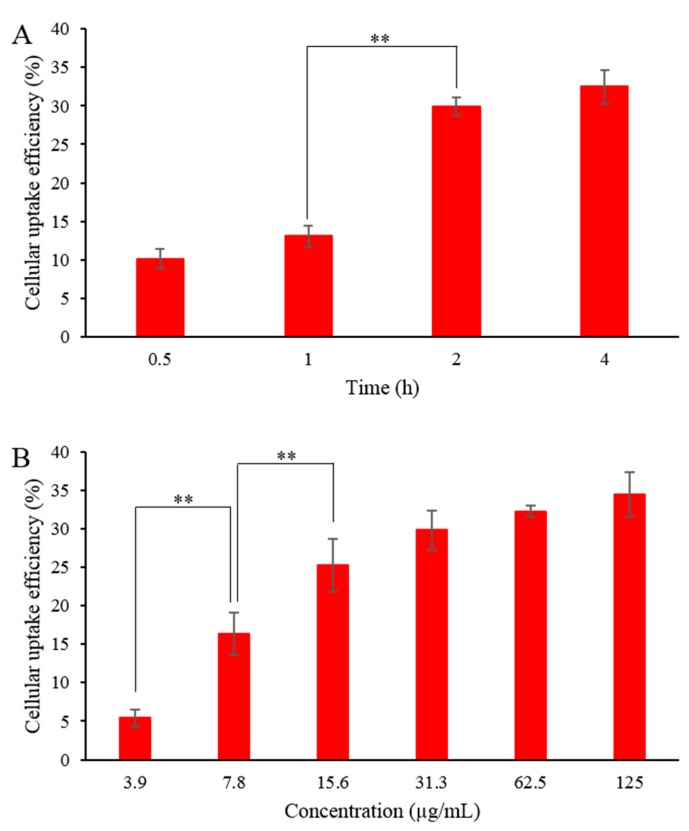
Cellular uptake profile of fDMP2 by HT-29 cells as a function of (**A**) incubation time and (**B**) concentration over an incubation period of 2 h (mean ± SD, *n* = 3); ** indicates *p* < 0.01 between the two time intervals.

The cellular uptake efficiency of fDMP2 by HT-29 cells was determined after 0.5, 1, 2, and 4 h of incubation with their respective IC_50_ concentrations (Figure 8A). The uptake efficiency of the samples showed a time-dependent effect that was directly proportional to the incubation time. The maximum cellular uptake efficiency of fDMP2 (32.5%) occurred by 4 h of incubation, with a marked increase (factor of 1.29) from 1 h to 2 h. Wang et al. [34] also reported that wheat germ agglutinin-conjugated nanoparticles showed a time-dependant effect and that a longer incubation time was needed in order to achieve a therapeutic level of the intracellular drug concentration. This prolonged time was essential for nanoparticles to release the drug in the cytoplasm. Therefore, 2 h was used herein as the optimum duration for cellular uptake.

The cellular uptake efficiency increased with increasing concentrations of fDMP2 (Figure 8B), illustrating a dose-dependent effect. Tukey’s HSD *post hoc* test showed a significant increase (*p* < 0.01) from 3.9 µg/mL to 15.6 µg/mL, with no significant difference for concentrations higher than 15.6 µg/mL. Therefore, the IC_50_ of fDMP2 (52.9 µg/mL) (which was >15.6 µg/mL) was suitable for fluorescent and confocal imaging.

**Figure 9:**
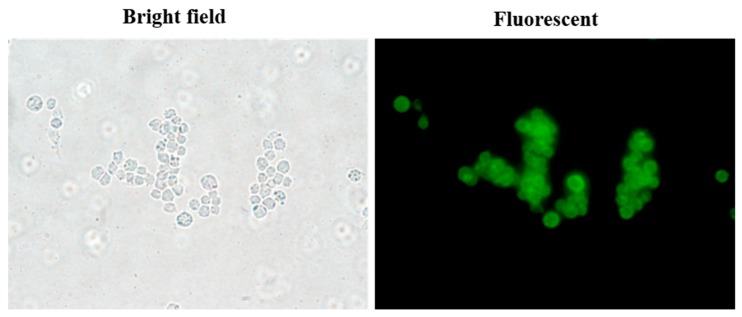
Cellular uptake of fDMP2 by HT-29 cells (2 h) under 40,000 × magnifications illustrated by fluorescence microscopy.

The cellular internalisation of Alexa Fluor 488 fWGA-labelled fDMP2 was studied using fluorescence microscopy (Figure 9). Cellular uptake of fDMP2 was confirmed when the incubated cells exhibited green fluorescence. Confocal imaging was further conducted to study the mechanism of cellular uptake of fDMP2.

**Figure 10:**
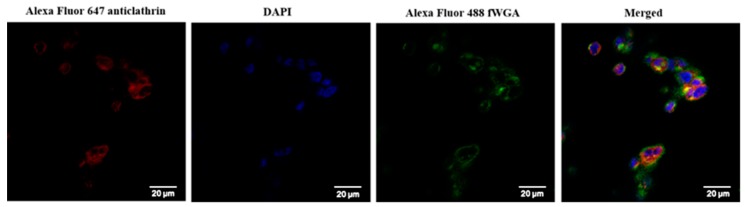
Colocalisation of clathrin for fDMP2 in HT-29 cells; localisation of clathrin, nucleus, nanoparticles, and the merged image in HT-29 cells.

HT-29 cells were incubated with IC_50_ concentrations of the nanoparticles (fDMP2) (green colour) for 2 h (optimum condition), treated with clathrin antibodies (red colour), and stained with DAPI (blue colour) (Figure 10). The red represents the acidic lysosomal compartments (endolysosomes), and the blue represents the nuclei. The nanoparticles (green) were mainly present in the cytosol with some perinuclear distribution. The integrated image indicates that colocalisation of the nanoparticles and clathrin very likely occurred, as a yellow colour is visible due to overlapping of the green of fWGA-conjugated nanoparticles and the red of the clathrin stain. This result implies that the cellular uptake mechanism of the nanoparticles is possibly clathrin-mediated endocytosis [34]. Internalisation of nanoparticles by HT-29 cells can occur through conjugation of WGA to its substrates, which were N-acetylglucosamine and sialic acid on the clathrin antibody-treated cell membrane. Subsequently, uptake of nanoparticles into the acidic lysosomal compartments possibly takes place via a consecutive process of cyto-adhesion and cyto-invasion [34,81,82].

## 4. Conclusions

pH-sensitive and reduction-responsive thiolated sodium alginate derivative nanoparticles conjugated with fWGA were successfully developed for delivery of DTX to colon cancer cells. Creation of the nanoparticles began with synthesis of MPA with different ratios of sodium alginate to 3-mercaptopropionic acid. MP disulfide cross-linked nanoparticles were successfully prepared via the self-assembly technique assisted by air oxidation and sonication. DTX was successfully loaded into MP nanoparticles (DMP1, DMP2, DMP3, and DMP5), and DMP2 was found to be the most promising formulation with the highest DTX loading efficiency (17.8%). Therefore, it was chosen for fWGA surface conjugation, and fDMP2 had an efficiency of 14.1%. In the in vitro drug release experiment (dialysis technique) under simulated gastrointestinal conditions, cumulative drug release was 48.6%. In DLS analysis, the MD of the nanoparticles was 289 nm, with a PDI of 0.3 and zeta potential of −2.2 mV.

In vitro cytotoxicity analysis of the nanoparticles against HT-29 and L929 cells showed that fDMP2 had IC_50_ values of 52.9 µg/mL for HT-29 cells and 201.6 µg/mL for L929 cells. These results show selectivity towards HT-29 cells over L929 cells, and this selectivity is promising for the delivery of anticancer drugs to colon cancer cells. fDMP2 had a cellular uptake efficiency of 29.9% after 2 h of incubation. Both fluorescence and confocal imaging showed possible internalisation of fDMP2 by HT-29 cells. In conclusion, fDMP2 is a promising carrier for controlled and sustained release of anticancer drugs to colon cancer cells.

## Figures and Tables

**Table 1:** MD, PDI, and zeta potential values of blank nanoparticles (MP1–5) and DTX-loaded nanoparticles (DMP1–5) (mean ± SD, *n* = 3).

Sample	MD (nm)	PDI	Zeta Potential (mV)
MP1	195 ± 7	0.46 ± 0.02	−2.1 ± 6.7
MP2	204 ± 3	0.14 ± 0.01	−49.1 ± 0.7
MP3	211 ± 4	0.40 ± 0.05	−9.7 ± 1.0
MP5	245 ± 8	0.20 ± 0.02	−54.7 ± 1.8
DMP1	244 ± 20	0.35 ± 0.04	−35.7 ± 2.7
DMP2	219 ± 25	0.48 ± 0.17	−38.6 ± 5.8
DMP3	229 ± 14	0.35 ± 0.09	−28.1 ± 2.2
DMP5	212 ± 5	0.55 ± 0.09	−17.7 ± 1.2

**Table 2:** MD, PDI, and zeta potential values of nanoparticles before and after conjugation with fWGA (mean ± SD, *n* = 3).

Sample	MD (nm)	PDI	Zeta Potential (mV)
MP2 (Before)	204 ± 3	0.14 ± 0.01	−49.1 ± 0.7
fMP2 (After)	274 ± 3	0.29 ± 0.02	−15.8 ± 1.5
DMP2 (Before)	219 ± 25	0.48 ± 0.17	−38.6 ± 5.8
fDMP2 (After)	289 ± 10	0.30 ± 0.02	−2.2 ± 2.4

**Table 3:** MD and PDI values of (**A**) MP1–5 and (**B**) fMP2 and fDMP2 nanoparticles before and after storage at pH 7.4 for 30 d (mean ± SD, *n* = 3).

**(A)** **Sample**	**Before Storage**		**After Storage**	
	**MD (nm)**	**PDI**	**MD (nm)**	**PDI**
MP1	191 ± 18	0.62 ± 0.03	213 ± 80	0.58 ± 0.18
MP2	226 ± 23	0.31 ± 0.03	253 ± 5	0.38 ± 0.12
MP3	226 ± 32	0.31 ± 0.03	259 ± 28	0.48 ± 0.22
MP5	233 ± 72	0.33 ± 0.02	257 ± 23	0.32 ± 0.13
**(B)** **Sample**	**Before Storage**		**After Storage**	
	**MD (nm)**	**PDI**	**MD (nm)**	**PDI**
fMP2	272 ± 11	0.29 ± 0.01	273 ± 8	0.28 ± 0.03
fDMP2	289 ± 10	0.3 ± 0.02	303 ± 18	0.29 ± 0.07

**Table 4:** Reduction response study results of (**A**) MP and (**B**) fMP2 nanoparticles (mean ± SD, *n* = 3); ** (*p* < 0.01) and * (*p* < 0.05) show significant differences between the samples.

**(A)** **Sample**	**GSH (0)**		**GSH (10 µM)**		**GSH (10 mM)**	
	**MD (nm)**	**PDI**	**MD (nm)**	**PDI**	**MD (nm)**	**PDI**
MP1	191 ± 18 *	0.62 ± 0.03	232 ± 46	0.67 ± 0.04	289 ± 31 *	0.67 ± 0.04
MP2	226 ± 23 **	0.31 ± 0.03	237 ± 22	0.42 ± 0.01	393 ± 39 **	0.46 ± 0.01
MP3	226 ± 32 **	0.31 ± 0.03	306 ± 17 **	0.36 ± 0.03	394 ± 12 **	0.43 ± 0.06
MP5	233 ± 72 *	0.33 ± 0.02	293 ± 35	0.32 ± 0.02	374 ± 12 *	0.43 ± 0.03
**(B)** **Sample**	**GSH (0)**		**GSH (10 µM)**		**GSH (10 mM)**	
	**MD (nm)**	**PDI**	**MD (nm)**	**PDI**	**MD (nm)**	**PDI**
fMP2	274 ± 26 **	0.29 ± 0.02	299 ± 21	0.29 ± 0.06	559 ± 34 **	0.73 ± 0.03

**Table 5:** Mathematical models and parameters based on fDMP2 release data.

pH	Zero-order	First-order	Higuchi’s	Hixson-Crowell’s	Korsmeyer-Peppas’s		Kopcha’s	
	R^2^	R^2^	R^2^	R^2^	R^2^	*n*	R^2^	*A*/*B*
1.0	0.56	0.58	0.83	0.02	0.58	0.14	0.90	6.28
7.4	0.86	0.85	0.89	0.86	0.97	−1.14	0.93	2.44
6.0	0.85	0.86	0.86	0.86	0.82	0.64	0.62	6.46

## Supplementary Materials

The following are available online at https://www.mdpi.com/1999-4923/12/1/38/s1, Figure S1: The calibration curve of DTX, Figure S2: The calibration curves of DTX in pH (a) 1.0, (b) 7.4 and (c) 6.0 media. All authors have read and agreed to the published version of the manuscript.
