# Supplementary material for: Docetaxel-Loaded Disulfide Cross-Linked Nanoparticles Derived from Thiolated Sodium Alginate for Colon Cancer Drug Delivery
Source: Pharmaceutics. 2020 Jan 2;12(1):38. doi: 10.3390/pharmaceutics12010038 (PMC7023491; doi:10.3390/pharmaceutics12010038)
Supplement: Supplementary file 1 [file pharmaceutics-12-00038-s001.pdf]

pharmaceutics

Supplementary Materials: Docetaxel-Loaded Disulfide Cross-Linked Nanoparticles Derived from Thiolated Sodium Alginate for Colon Cancer Drug Delivery

Hock Ing Chiu, Asila Dinie Ayub, Siti Nur Aishah Mat Yusuf, Noorfatimah Yahaya, Erazuliana Abbd Kadir and Vuanghao Lim

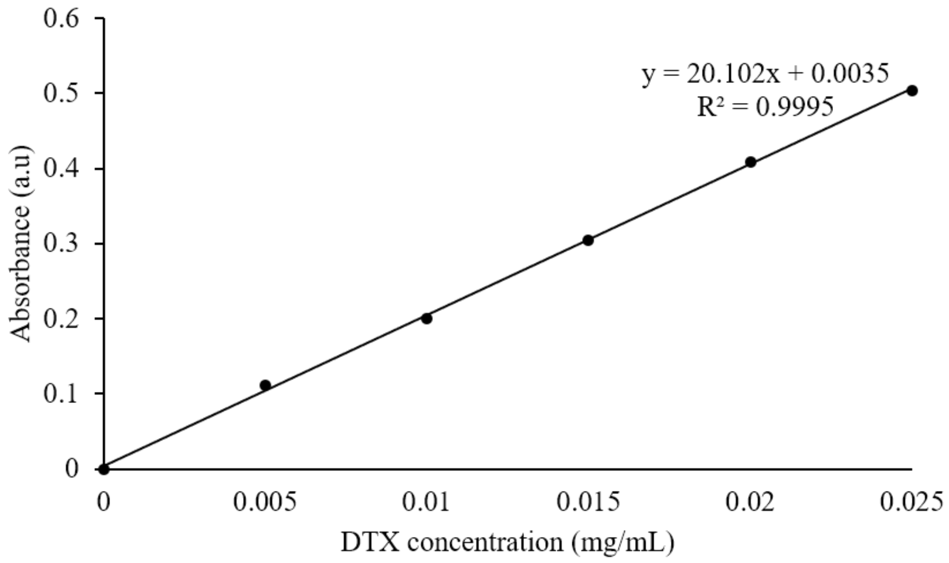

Figure S1. The calibration curve of DTX.

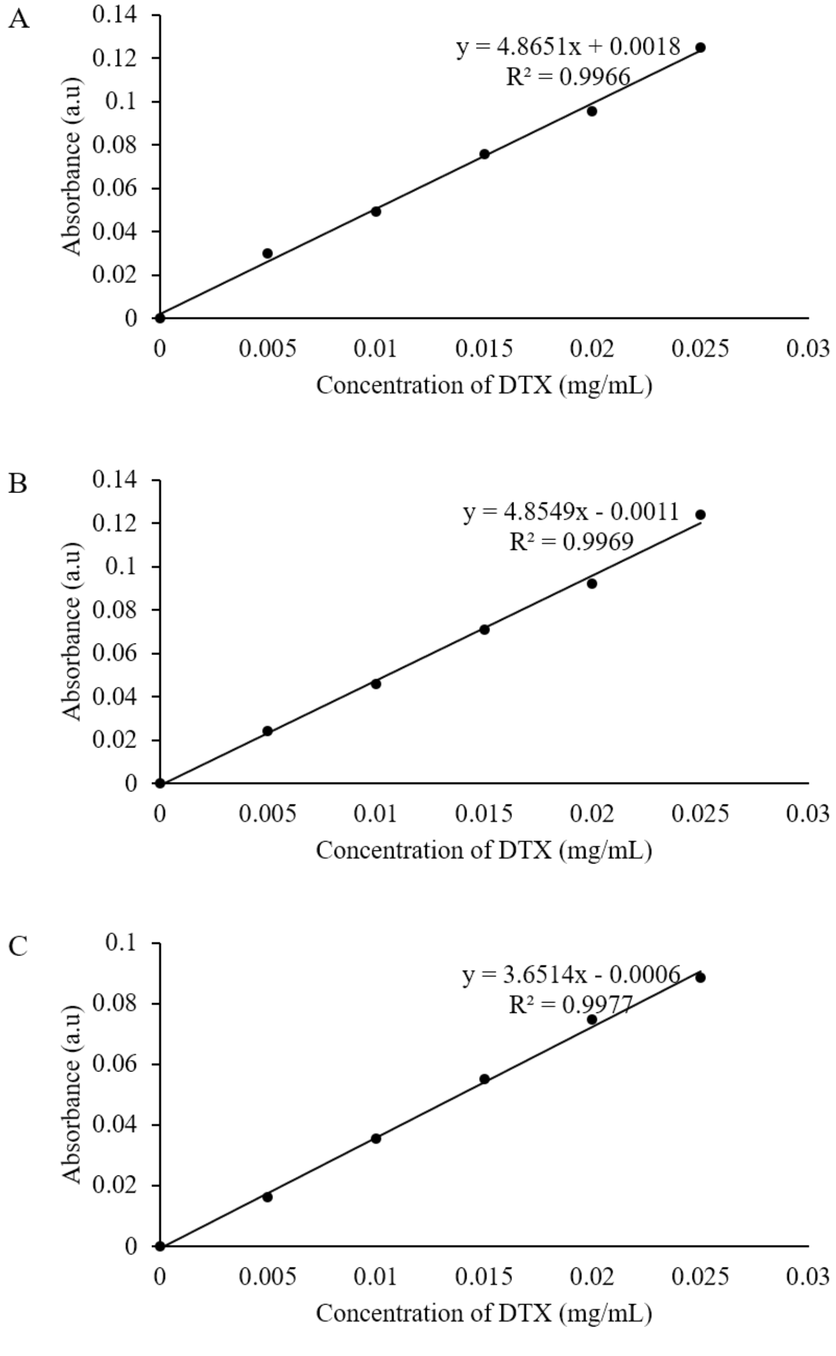

Figure S2. The calibration curves of DTX in pH (**a**) 1.0, (**b**) 7.4 and (**c**) 6.0 media.
